# Effects and safety of Buyang-Huanwu Decoction for the treatment of patients with acute ischemic stroke: A protocol of systematic review and meta-analysis

**DOI:** 10.1097/MD.0000000000020534

**Published:** 2020-06-05

**Authors:** Chao Jiang, Yong-cheng Xu, Wen Zhang, Wen Pan, Xu Chao

**Affiliations:** a The Third Department of Neurology, The Second Affiliated Hospital of Xi’an Medical University, Xi’an; b Department of Emergency, Longhua Hospital Shanghai University of Traditional Chinese Medicine; c Department of Vascular Disease, Shanghai Traditional Chinese Medicine Integrated Hospital, Shanghai; d Department of Emergency, Ankang Hospital of Traditional Chinese Medicine, Ankang; e Department of Scientific Research, The Second Affiliated Hospital of Shaanxi University of Chinese Medicine, Xianyang; f The College of Basic Medicine Sciences, Shaanxi University of Chinese Medicine, Xi’an, Shaanxi, PR China.

**Keywords:** acute ischemic stroke, Buyang-Huanwu Decoction, effects, randomized controlled trial, safety

## Abstract

**Background:**

We designed this study to assess the effects and safety of Buyang-Huanwu Decoction (BYHWD) for the treatment of patients with acute ischemic stroke (AIS).

**Methods:**

Electronic databases of Cochrane Library, EMBASE, MEDLINE, CINAHL, PsycINFO, Scopus, Allied and Complementary Medicine Database, VIP Database, and China National Knowledge Infrastructure will be comprehensively and systematically searched from initial time of each electronic database to the present without limitations of language and publication status. Randomized controlled trials on BYHWD alone against any other interventions for the treatment of AIS will be included. All process of study selection, data collection, and methodological quality assessment will be independently undertaken by 2 investigators. Cochrane risk of bias tool and RevMan 5.3 software will be utilized for the performance of methodological quality assessment and statistical analysis, respectively.

**Results:**

This study will summarize most recent high quality evidence on investigating the effects and safety of BYHWD alone against any other interventions for the treatment of patients with AIS.

**Conclusions:**

The findings of this study will provide helpful evidence for the clinical practice for patients with AIS using BYHWD, as well as the relevant future researches.

Study registration number: INPLASY202040169.

## 1 Introduction

Acute ischemic stroke (AIS) is the most common type of stroke,^[1–3]^ which often result in high mortality and morbidity in adult population.^[4–7]^ It has been reported that about 13 million cases are identified worldwide each year, and about more than 5 million deaths occurred.^[8]^ Previous study has reported that about 1,900,000 brain cells are lost, and 14,000,000,000 nerve connections are destroyed every minute in patients with AIS.^[9]^ Thus, it is very important to treat AIS timely and effectively. Although lots of managements have utilized to treat AIS, the efficacy is still limited.

Fortunately, a variety of studies reported that Buyang-Huanwu Decoction (BYHWD) has been used for the treatment of patients with AIS.^[10–23]^ However, there is no systematic review exploring the effect and safety of BYHWD for AIS. Thus, this study is the first one to systematically assess the effects and safety of BYHWD for patients with AIS.

## 2 Methods

### 2.1 Study registration

We have registered the present study on INPLASY202040169. It has been carried out based on the guidelines of Preferred Reporting Items for Systematic Review and Meta-Analysis Protocols Statement.^[24]^

### 2.2 Criteria for including studies

#### 2.2.1 Types of studies

Only published or unpublished randomized controlled trials (RCTs) will be included, which explored the effects and safety of BYHWD for the treatment of patients with AIS.

#### 2.2.2 Types of patients

The patients will be adults (18 years old or more) who were diagnosed as AIS. No limitations on country, ethnicity, gender, economic status, and educational background will be implemented.

#### 2.2.3 Types of interventions

All types of BYHWD for the treatment of patients with AIS will be selected as an experimental intervention. However, patients who received the combination of BYHWD with other managements are not qualified in this study.

In the control group, patients could undergo any treatments, but not any forms of BYHWD.

#### 2.2.4 Types of outcome measurements

Primary outcomes are the proportion of recurrent ischemic stroke, symptomatic intracerebral haemorrhage, and the number of all-cause mortality.

Secondary outcomes are functional improvement, as measured by the validated Barthel index or other scales; quality of life, as assessed by the 36-Item Short Form Health Survey; and frequency and severity of adverse events.

### 2.3 Data sources and search

We will search Cochrane Library, EMBASE, MEDLINE, CINAHL, PsycINFO, Scopus, Allied and Complementary Medicine Database, VIP Database, and China National Knowledge Infrastructure for related trials published from initial time of each electronic database to the present without any language and publication status limitations. Any randomized controlled trials which focus on assessing the effects and safety of BYHWD alone against any other interventions for the treatment of AIS will be included. The Cochrane Library search strategy is presented in Table 1. We will adapt similar search strategies to the other electronic databases.

**Table 1:**
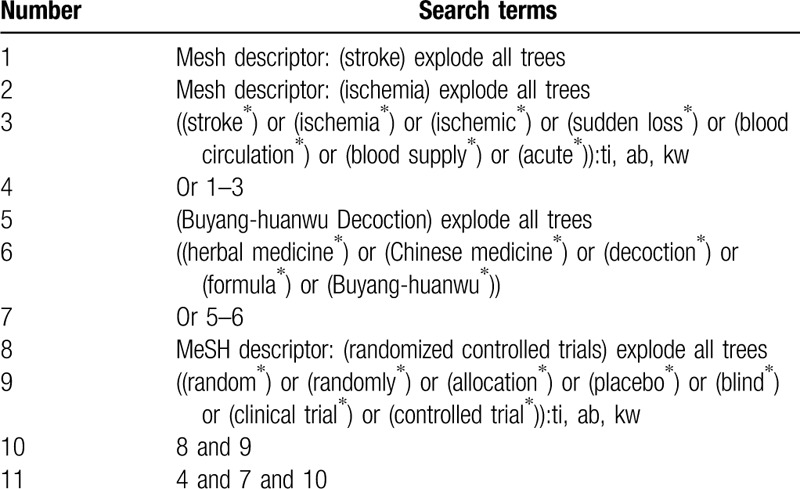
Search strategy for Cochrane Library.

In addition to the electronic databases, we will also search other sources, such as websites of clinical trial registry, dissertations, conference proceedings, and reference lists of included trials or related reviews.

### 2.4 Data collection and analysis

#### 2.4.1 Study selection

Two investigators will independently check each title/abstract of all records searched and identify whether the trials fulfill the edibility criteria as described and designed in this protocol. All duplicated and irrelevant studies will be removed. Full papers of all remaining records will be carefully read against all inclusion criteria. Reasons for all excluded studies will be noted at different stages. Any disagreements between 2 investigators will be solved by discussion with a third investigator if necessary. We will present the selection of study in details in a diagram chart.

#### 2.4.2 Data extraction

Two investigators will extract the essential data from the included trials, independently and respectively. Any different ideas between both of them will be resolved by consultation with a third investigator. The included data consists of study characteristics and methodology (such as first author, publication date, study setting, study design, randomization, study duration, follow-up duration, withdrawals, etc); participant characteristics (such as age, gender, ethnicity, diagnosis, eligibility criteria, etc); details of interventions and comparators (types of delivery, frequency, duration of delivery, dosage, etc), outcomes (all primary and secondary outcomes, safety, etc), funding information and conflict of interests. If reported data of included trials are insufficient or missing, we will contact original corresponding authors to request them.

#### 2.4.3 Risk of bias assessment

Two investigators will independently appraise the risk of bias for each included trial using Cochrane risk of bias tool. Any divergences will be solved by a third investigator through consultation. This tool has 7 domains, and each item is evaluated as high, unclear, or uncertain risk of bias.

#### 2.4.4 Subgroup analysis

Subgroup analysis will be undertaken to search potential causes of heterogeneity in study characteristics, participant characteristics, study methods, intervention and controls, and outcomes.

#### 2.4.5 Sensitivity analysis

Sensitivity analysis will be carried out to identify the reliability and stability of aggregation results through eliminating trials with high risk of bias.

#### 2.4.6 Publication bias

If over 10 trials are included, we will construct a funnel plot and Egger regression test to check possible publication bias.^[25,26]^

#### 2.4.7 Evidence evaluation

We will assess all the evidence according to the criteria of Recommendations Assessment, Development, and Evaluation.^[27]^ It covers 5 aspects, and the quality evidence of each outcome will be graded as high, moderate, low, and very low.

### 2.5 Data synthesis

In this study, all extracted data will be synthesized and analyzed using RevMan 5.3 software. In addition, we will perform a systematic review and meta-analysis if the collected data is judged to be similar adequate to make a certain result that is meaningful. We will express continuous values using mean difference or standardized mean difference and 95% confidence intervals (CIs), and dichotomous values utilizing risk ratio and 95% CIs. Statistical heterogeneity across included trials will be checked using *I*^2^ statistics. *I*^2^ ≤ 50% shows acceptable heterogeneity, and a fixed-effect model is used. If sufficient data on the same outcome measurement are collected, a meta-analysis will be conducted. *I*^2^ > 50% presents significant heterogeneity, and a random-effect model will be employed. Subgroup analysis will be performed to explore the causes of substantial heterogeneity. If the data is deemed not to be pooled, the results will be elaborated as a narrative summary.

## 3 Discussion

The literature shows that an increasing number of clinical trials have tested the effect and safety of BYHWD for AIS. So far, no systematic review has been performed to appraise the comparative effect and acceptability of BYHWD for AIS. Thus, it is necessary to develop a systematic and comprehensive study that examines the potential effect and safety of BYHWD for the treatment of AIS. The findings of this study will provide scientific evidence for clinician and future research who work in this field of knowledge.

## Author contributions

**Conceptualization:** Chao Jiang, Xu Chao.

**Data curation:** Wen Zhang, Wen Pan.

**Formal analysis:** Chao Jiang, Wen Pan, Xu Chao.

**Funding acquisition:** Chao Jiang.

**Investigation:** Wen Pan, Xu Chao.

**Methodology:** Chao Jiang, Wen Zhang.

**Project administration:** Wen Pan, Xu Chao.

**Resources:** Chao Jiang, Yong-cheng Xu, Wen Zhang.

**Software:** Chao Jiang, Yong-cheng Xu.

**Supervision:** Wen Pan, Xu Chao.

**Validation:** Chao Jiang, Yong-cheng Xu, Wen Zhang, Wen Pan, Xu Chao.

**Visualization:** Chao Jiang, Wen Zhang, Wen Pan, Xu Chao.

**Writing – original draft:** Chao Jiang, Yong-cheng Xu, Wen Pan, Xu Chao.

**Writing – review & editing:** Chao Jiang, Yong-cheng Xu, Wen Zhang, Wen Pan, Xu Chao.
